# Implication of protein post translational modifications in gastric cancer

**DOI:** 10.3389/fcell.2025.1523958

**Published:** 2025-02-04

**Authors:** Houji Song, Mingze Zhang, Chengwang Guo, Xi Guo, Yuqi Ma, Yuntao Ma

**Affiliations:** ^1^ The First Clinical Medical College, Gansu University of Traditional Chinese Medicine, Lanzhou, China; ^2^ Department of General Surgery, Gansu Provincial Hospital, Lanzhou, China

**Keywords:** gastric cancer, ubiquitination, phosphorylation, acetylation, glycosylation, methylation, lactylation, SUMOylation

## Abstract

Gastric cancer (GC) is one of the most common and highly lethal malignant tumors worldwide, and its occurrence and development are regulated by multiple molecular mechanisms. Post-translational modifications (PTM) common forms include ubiquitylation, phosphorylation, acetylation and methylation. Emerging research has highlighted lactylation and glycosylation. The diverse realm of PTM and PTM crosstalk is linked to many critical signaling events involved in neoplastic transformation, carcinogenesis and metastasis. This review provides a comprehensive overview of the impact of PTM on the occurrence and progression of GC. Specifically, aberrant PTM have been shown to alter the proliferation, migration, and invasion capabilities of GC cells. Moreover, PTM are closely associated with resistance to chemotherapeutic agents in GC. Notably, this review also discusses the phenomenon of PTM crosstalk, highlighting the interactions among PTM and their roles in regulating signaling pathways and protein functions. Therefore, in-depth investigation into the mechanisms of PTM and the development of targeted therapeutic strategies hold promise for advancing early diagnosis, treatment, and prognostic evaluation of GC, offering novel insights and future research directions.

## 1 Introduction

Gastric cancer (GC) is a malignant tumor originating from the gastric mucosa, usually developed from glandular cells in the stomach (Smyth et al., 2020). GC is a public health problem worldwide. Exposition to *Helicobacter pylori* infection and dietary risk factors for GC shape the epidemiology of this disease (Tirado-Hurtado et al., 2019; Parsonnet et al., 1991; Wang et al., 2014). The incidence rate of GC varies significantly worldwide, especially in East Asia (such as China, Japan and South Korea) (Lopez et al., 2023; Davis and Sano, 2001; Bray et al., 2015). GC is the fifth most common cancer and the fifth most common cause of cancer death globally (Bray et al., 2024).

**FIGURE 1:**
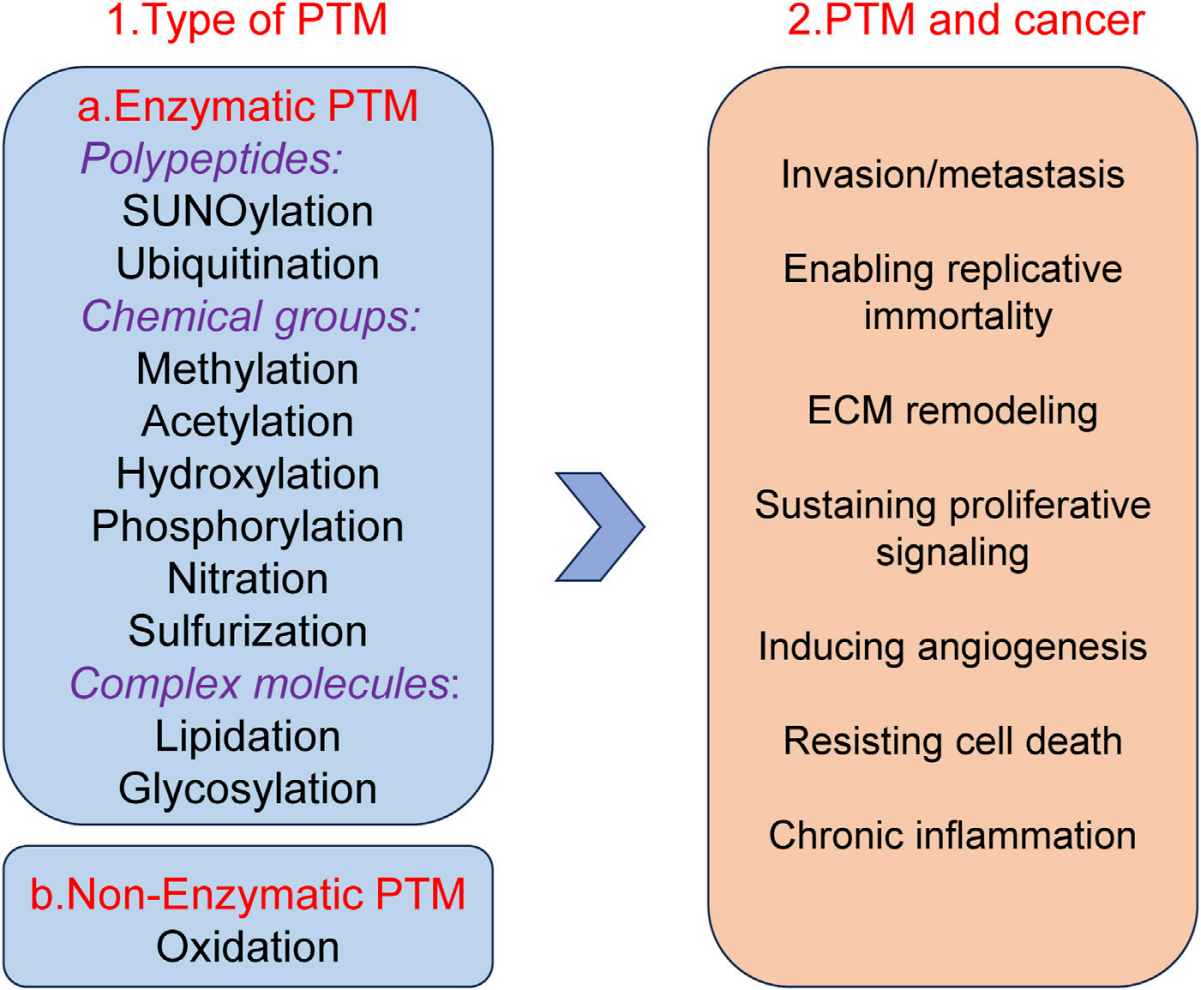
Well-known examples of PTM and Pathogenesis implications of PTM in cancer.

Post translational modifications (PTM) refer to a series of chemical modifications that occur after protein synthesis is completed (Deribe et al., 2010). Common PTM include phosphorylation, acetylation, glycosylation, ubiquitination, methylation, lactylation, etc., (Khoury et al., 2011; Pan and Chen, 2022). PTM can affect cell proliferation, apoptosis, invasion and metastasis by regulation protein activity, stability, localization and interactions with other molecules (Lee et al., 2023; Deribe et al., 2010; Vu et al., 2018; Pienkowski et al., 2023). Different types of PTM together form a complex network for protein functional regulation (Figure 1). In summary, PTM of proteins play a crucial role in biological processes. Therefore, studying PTM is crucial for understanding cell biology and developing new therapeutic strategies.

The recent research showed that the occurrence and development of GC are closely related to protein PTM (Paska and Hudler, 2015; Tan et al., 2007; Ramesh et al., 2023). Understanding the role of PTM of proteins in the occurrence and development of GC plays an important role in the treatment and prognosis of GC. Nowadays, there are many FDA approved targeted drugs on PTM (Table 1).

**TABLE 1:** PTM types of targeted drugs.

Type of modification	Drugs	Status
Ubiquitination	Bortezomib (Hideshima et al., 2011)	FDA approved for MM, MCL, NSCLC and PAAD
	Carfilzomib (Hajek et al., 2012)	FDA approved for MM
	Thalidomide (Bartlett et al., 2004; Ito et al., 2010)	FDA approved for MM
	Lenalidomide (Lopez-Girona et al., 2012; Syed, 2017)	FDA approved for MM
	Pomalidomide (Lopez-Girona et al., 2012)	FDA approved for MM
	PRIMA (Bykov et al., 2017)	FDA approved for LIHC and PAAD
	Erioflorin (Jaffry and Wells, 2023)	Preclinical/research
	b-AP15 (Morgan et al., 2023)	Preclinical/research
Phosphorylation	Afatinib (Fukuda and Okuma, 2024)	FDA approved for NSCLC
	Aumolertinib (Lu et al., 2022)	NMPA approved for NSCLC
	Dacomitinib (Hosamani et al., 2024)	FDA approved for NSCLC, BRCA and MM
	Erlotinib (Dowell et al., 2005)	FDA approved for NSCLC and PAAD
	Cetuximab (Bokemeyer et al., 2024)	FDA approved for CRC and HNL
	Copanlisib (Deshpande and Munoz, 2022)	FDA approved for FL
	TNO155 (Chai et al., 2024)	Preclinical/research
	SM08502 (Martin Moyano et al., 2020)	Preclinical/research
	Ramucirumab (Lin et al., 2024)	FDA approved for GC
Acetylation	Vorinostat (Wawruszak et al., 2021)	FDA approved for CTCL
	Belinostat (O’Connor et al., 2024)	FDA approved for PTCL
	Panobinostat (Sivaraj et al., 2017)	FDA approved for MM
	Chidamide (Li et al., 2019)	NMPA approved for PTCL
	Romidepsin (Li et al., 2019; Argnani et al., 2017)	FDA approved for MM and CTCL
Glycosylation	gPD-L1 (Lee et al., 2023)	Preclinical/research
Methylation	5’-azacytidine (Wang et al., 2024c)	FDA approved for AML and CMML
	Decitabine (Dhillon, 2020)	FDA approved for AML, CMML and GBM
	Valemetostat (Zinzani et al., 2024)	Phase II for PTCL
	Capecitabine (Hameed and Cassidy, 2011)	FDA approved for CRC and GC

MM, multiple myeloma; MCL, mantle cell lymphoma; NSCLC, non-small-cell lung cancer; PAAD, pancreatic adenocarcinoma; LIHC, liver hepatocellular carcinoma; BRCA, breast invasive carcinoma; CRC, colorectal cancer; HNC, head and neck cancer; FL, follicular lymphoma; GC, gastric cancer; CTCL, cutaneous T-cell lymphoma; PTCL, peripheral T cell lymphoma; AML, acute myeloid leukemia; CMML, chronic myelomonocytic leukemia; NMPA, National Medical Products Administration.

Several PTM-targeted therapies have already been approved by the FDA. These therapies are characterized by their high specificity, enabling precise modulation of critical signaling pathways while minimizing off-target effects. Additionally, their dynamic and reversible nature provides greater flexibility and adaptability in therapeutic applications. However, PTM-targeted drugs also face certain limitations. The intricate biological mechanisms underlying PTM complicate target identification and drug design. Furthermore, the high spatial and temporal specificity of certain PTM may restrict the applicability of these drugs across different tissues or diseases.

The purpose of this review is to outline the role of common protein PTM in GC.

## 2 The PTM of GC

### 2.1 Ubiquitination

**FIGURE 2:**
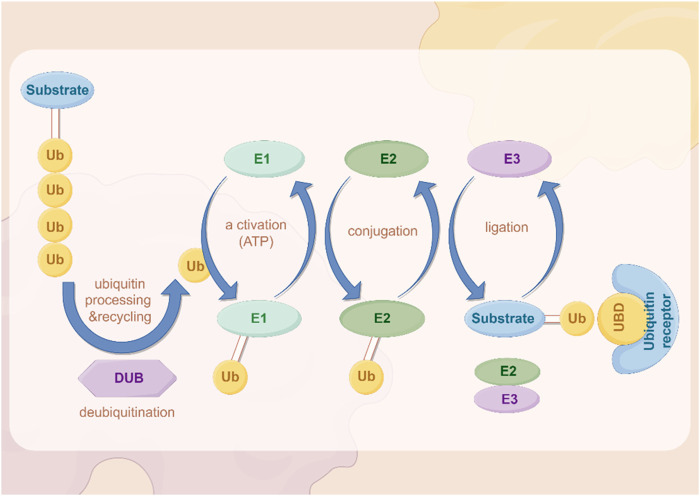
The process of ubiquitination. The figure was drawled by Figdraw (www.figdraw.com/#).

In recent years, important results have been achieved regarding the role of ubiquitination. Ubiquitin is a highly conserved small molecule protein that exists in all eukaryotic cells (Popovic et al., 2014). It is composed of 76 amino acids and has a molecular weight of approximately 8.5 kDa (Popovic et al., 2014; Cockram et al., 2021). Ubiquitination regulation is a dynamic process regulated by both ubiquitinases and deubiquitinases (Figure 2). It is a PTM process in which ubiquitin is covalently attached to target proteins through three main steps: activation, conjugation, and ligation (Dikic and Schulman, 2023). First, the E1 ubiquitin-activating enzyme activates ubiquitin via ATP hydrolysis, forming an E1-ubiquitin thioester intermediate (Dikic and Schulman, 2023). Subsequently, the activated ubiquitin is transferred to the E2 ubiquitin-conjugating enzyme (Dikic and Schulman, 2023). Finally, the E3 ubiquitin ligase recognizes specific target proteins and catalyzes the transfer of ubiquitin from the E2-ubiquitin complex to a lysine residue on the target protein, resulting in ubiquitinated proteins (Dikic and Schulman, 2023). Through repeated cycles, polyubiquitin chains can be formed, which regulate various biological functions such as protein degradation, signal transduction, and subcellular localization (Dang et al., 2021; Li et al., 2021; Sampson et al., 2023; Qiu et al., 2023).

Ubiquitin complexes can be degraded by ubiquitinases, and this process is reversible, with deubiquitinases (DUBs) removing ubiquitin molecules from target proteins (Harrigan et al., 2018; Dewson et al., 2023). DUBs recover ubiquitin by hydrolyzing the heteropeptide bond between ubiquitin and target proteins, regulating protein degradation and cellular function (Mevissen and Komander, 2017). This step plays an important role in maintaining cellular homeostasis and regulating protein degradation balance.

In the occurrence and progression of GC, abnormalities in the ubiquitination system can lead to the degradation of tumor suppressor genes and excessive activation of oncogenes, thereby promoting the occurrence and development of tumors (Wang D. et al., 2022; Liu et al., 2020; Sokolova and Naumann, 2021; Li K. Q. et al., 2024; Hou and Deng, 2015; Sun et al., 2020b).

The ubiquitination system can also promote tumor growth by regulating the stability of certain oncogenes (Popovic et al., 2014). While the roles of DUBs in GC have been recently reviewed (An et al., 2022), here, some E3 ubiquitin ligases may enhance the function of oncogenes by protecting them from degradation, thereby promoting the growth and metastasis of GC. For example, studies have shown that the ubiquitination system can promote the proliferation and survival of GC cells by regulating cellular signaling pathways such as the NF - κB pathway (Yang W. et al., 2023).The documented roles of these proteins in GC are summarized in Table 2.

**TABLE 2:** Ubiquitin E3 ligases and processes they influence in GC.

E3 Ligase	Brief biological mechanism	References
BMI1	Mel-18 downregulates BMI1, influence cell migration and metastasis through the p16 and AKT-dependent growth regulatory pathways	Zhang et al. (2010)
	Bmi-1-induced miR-27a and miR-155 promote tumor metastasis and chemoresistance by targeting RKIP	Li Y. et al. (2020)
	The inhibitory effect of human DEFA5 in growth of GC by targeting BMI1	Wu et al. (2021b)
	SOX9 promotes tumor progression through the axis BMI1-p21	Aldaz et al. (2020)
CAND1	GSTM3 promotes GC via CAND1/NRF2-KEAP1 signaling	Chen et al. (2022)
c-CBL	Beta-Elemene inhibits the metastasis of multidrug-resistant GC cells through miR-1323/Cbl-b/EGFR pathway	Deng et al. (2020)
	LncRNA MIR31HG controls the proliferation and metastasis of GC by c-CBL-mediated degradation of β-catenin	Peng et al. (2023)
COP1	COP1 promotes umorigenesis of GC by downregulation of CDH18 via PI3K/AKT signal pathway	Zhao et al. (2023a)
CRL4/Cdt2	Indisulam promotes the interaction between ZEB1 and DCAF15 to facilitate the migration of GC cells	Lu et al. (2023)
CUL4B	CUL4B promotes GC invasion and metastasis-involvement of upregulation of HER2	Qi et al. (2021)
FBXW7	The lncRNA BDNF-AS/WDR5/FBXW7 axis mediates ferroptosis in GC peritoneal metastasis by regulating VDAC3 ubiquitination	Huang et al. (2022b)
	ZC3H15 promotes GC progression by targeting the FBXW7/c-Myc pathway	Hou et al. (2022)
FBW7	PTBP1 mediates GC progression by upregulating USP28 and restricting FBW7-mediated ubiquitination of c-Myc	Ni et al. (2023)
MDM2	DHRS4-AS1 binds to DHX9 and recruits the E3 ligase MDM2, leading to the degradation of DHX9 to regulate apoptosis and cell proliferation in GC cells	Xiao et al. (2023)
RNF2	RASSF10/NPM/RNF2 axis promotes GC	Lakshmi Ch et al. (2021)
	Circ_0004104 Regulats the miR-539-3p/RNF2 Axis to promotes GC	Yue et al. (2021)
RNF6	RNF6 promotes GC progression by regulating CCNA1/CREBBP	Jiang et al. (2023)
SKP2	PHF5A facilitates the development and progression of GC through SKP2-mediated stabilization of FOS	Zhang et al. (2023f)
SOCS2	POU6F1 increase lncRNA-CASC2 transcription to regulate SOCS2/SLC7A11 signaling in GC	Wang et al. (2024b)
TRAF6	POU5F1 reduces the ubiquitination level of TRAF6 to promote GC	Yang et al. (2023b)
TRIM11	TRIM11-Axin1-β-catenin axis drive GC	Zhou et al. (2022)
TRIM25	HDSP interacts with MECOM to block TRIM25-mediated ubiquitination and degradation, resulting in MECOM accumulation and enhanced SPINK1 transcription	Chen et al. (2024b)
	JP3 regulates the TRIM25/SP1/MMP2 axis to inhibit angiogenesis in GC.	Chen et al. (2020)
β-TrCP	Disrupting the LNC942-MSI2-c-Myc axis promotes the treatment of GC	Zhu et al. (2022)
UHRF1	UPAT promotes GC cell progression via UHRF1	Liu et al. (2022)
HACE1	HACE1 regulates the ubiquitination of cyclin C, affecting cisplatin sensitivity in GC	Jiang et al. (2022)
HUWE1	HUWE1 mediates TGFBR2 ubiquitination to promote GC	He et al. (2021)
Nedd4	PHB2 promotes SHIP2 ubiquitination via NEDD4 to regulate AKT signaling in GC	Xu et al. (2024)
	MCCC2 interacts with NEDD4 to promote the ubiquitination and degradation of MCCC2 protein	He et al. (2024)
WWP2	WWP2 facilitating the ubiquitination and degradation of LATS1 to drive progression of GC	Zou et al. (2023)

The ubiquitination system plays a critical role in cell cycle regulation and DNA repair processes (Louie and Kurzrock, 2020; Dagar et al., 2023). Abnormal ubiquitination can lead to uncontrolled cell cycle and obstacles to DNA damage repair, thereby increasing the risk of GC. Research has shown that E3 ubiquitin ligase SKP2 can promote the degradation of cyclin inhibitor p27, leading to uncontrolled cell cycle, which is related to the development of GC (Wen et al., 2016; Ge et al., 2023).

Abnormalities in the ubiquitination system are also closely related to the resistance of GC patients to chemotherapy drugs (Gonzalez et al., 2023; Narayanan et al., 2020). GC cells promote the stability of anti-apoptotic proteins by upregulating specific ubiquitinases, thereby evading the effects of chemotherapy drugs (Niu et al., 2021; Xu et al., 2014).

In the tumor microenvironment of GC, ubiquitination regulates the expression and function of oncogenic genes, influencing the interactions between tumor cells and their surrounding microenvironment (Aichem and Groettrup, 2016). Additionally, ubiquitination modifications modulate immune evasion mechanisms, enabling cancer cells to evade recognition and attack by the host immune system, thereby promoting tumor progression and recurrence (Zhang C. et al., 2023). Furthermore, the association between ubiquitination and cancer treatment has become increasingly significant, particularly in chemotherapy and targeted therapies. Abnormal ubiquitination may affect the efficacy of therapeutic agents and contribute to the development of drug resistance in cancer cells, driving the advancement of personalized treatment strategies (Sun W. et al., 2022).

Due to the important role of the ubiquitination system in GC, targeted therapy targeting the ubiquitination process may become a new approach for treating GC. In summary, abnormalities in the ubiquitination system play a key role in the occurrence, progression, and drug resistance of GC. In depth research on the mechanism of ubiquitination and its specific regulatory pathways in GC can help discover new therapeutic targets and improve the prognosis of GC patients.

### 2.2 Phosphorylation

Protein phosphorylation is the most common and important in PTM (Zhang W. J. et al., 2023). Approximately 30% of the human proteome is phosphorylated, which is involved in almost all cellular life processes such as cell division, protein breakdown, signal transduction, gene expression regulation, and protein interactions (Li Y. et al., 2023; Singh et al., 2017). Many phosphorylation pathways, including MAPK, PI3K/Akt, tyrosine kinase, cadherin catenin complex, cyclin dependent kinase, NF -κB, TGF -β signaling, etc., which pathway play important roles in cancer development (Yuan et al., 2020; Cargnello and Roux, 2011; Fresno Vara et al., 2004; Koromilas and Mounir, 2013; Du and Lovly, 2018; Hubbard and Till, 2000; Le et al., 2019; Singh et al., 2017; Fischer et al., 2022; Karin and Ben-Neriah, 2000; Wang et al., 2023c; Zhang Q. et al., 2019).

**FIGURE 3:**
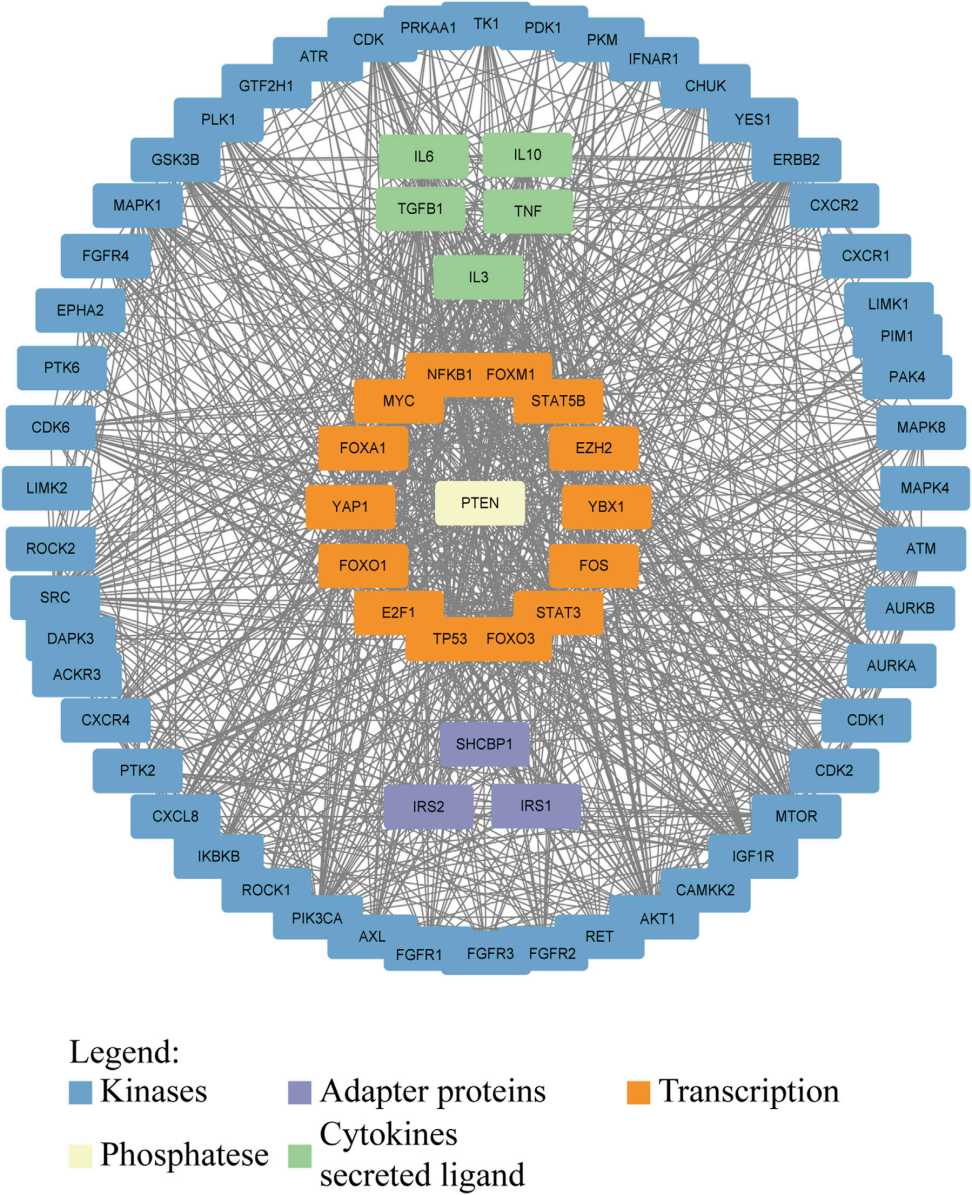
Interaction network of proteins involved in or affected by phosphorylation in GC. Kinases, adapter proteins, and transcription factors are shown to visualize the network that drives GC progression. Protein–protein interactions were downloaded from the STRING database (https://cn.string-db.org/) and visualized in Cytoscape.

Phosphorylation regulates many key molecules and signaling pathways associated with GC, and abnormal phosphorylation levels may promote the occurrence, progression, and metastasis of GC (Mun et al., 2019; Miao et al., 2023; Jiang et al., 2021). Cytoplasmic adapter proteins that become phosphorylated and activated downstream of many kinases are a link between kinases and other events of signaling cascades (Figure 3). Research has shown that phosphorylation of EGFR receptors activates downstream pathways (Cardoso et al., 2014; Zhang G. et al., 2023). In GC, p53 gene mutations often lead to ineffective phosphorylation regulation, further promoting the development of cancer (Yuan et al., 2022). The PI3K/AKT/mTOR signaling pathway is a key pathway that promotes cell proliferation, survival, and metabolism (Glaviano et al., 2023). In GC patients, key components of this pathway are often abnormally activated by phosphorylation, especially the excessive phosphorylation of AKT, which is associated with tumor proliferation and metastasis (Shen et al., 2023). The increase of AKT phosphorylation can not only inhibit cell apoptosis, but also promote protein synthesis and cell growth by affecting mTOR, further promoting the progression of GC (Wang C. et al., 2021; Zhong et al., 2023). Phosphorylation also plays an important role in regulating the activity of cell cycle proteins and apoptosis related proteins. In GC cells, abnormal phosphorylation levels can inhibit cell apoptosis and promote tumor cell survival (Rong et al., 2020). The invasion and metastasis of GC are one of the main reasons for poor prognosis in patients (Matsuoka and Yashiro, 2023). The signaling pathway regulated by phosphorylation plays a crucial role in cell movement, matrix degradation, and invasion processes. Abnormal phosphorylation of ERK can activate downstream molecules and enhance the migration and invasion ability of GC cells (Kim et al., 2024; Wu et al., 2010). Phosphorylation abnormalities are closely related to the resistance of GC to chemotherapy and targeted therapy (Wu et al., 2023c). Research has shown that GC cells can evade chemotherapy induced apoptosis by activating phosphorylation of key proteins on the PI3K/AKT pathway (Rong et al., 2020). The efficacy of drugs targeting EGFR in GC is also reduced due to resistance caused by phosphorylation activation (Cao et al., 2022). The documented roles of these kinases in GC are summarized in Table 3.

**TABLE 3:** Illustration of the mechanism of phosphatases in GC.

Phosphokinase	Brief biological mechanism	References
PKG	Regulates the polarization of macrophage M1 and influences the malignant progression of GC	Ma et al. (2024)
	PKG blocks activation in GC cells via Ser254 of PDGFRβ	Pang et al. (2022)
	ZEB1-upregulated protein PRTG induced promotes GC through the cGMP/PKG signaling pathway	Xiang et al. (2021)
AKT	Rps3 attenuates GC Lesions by promoting dendritic cells maturation via AKT/β-Catenin pathway	Li S. et al. (2024)
	LAMC2 regulates the proliferation, invasion, and metastasis of GC via PI3K/Akt signaling pathway	Cheng et al. (2024)
	TMEM65 promotes GC by targeting YWHAZ to activate PI3K-Akt-mTOR pathway	Shi et al. (2024)
PIM	Resveratrol suppresses GC cell proliferation and survival through inhibition of PIM-1 kinase activity	Kim et al. (2020)
CAMK	MicroRNA-135b/CAMK2D axis contribute to malignant progression of GC through EMT process remodeling	Huangfu et al. (2021)
MAPK	The VEGFA-Induced MAPK-AKT/PTEN/TGFβ signal pathway enhances progression and MDR in GC	Fang et al. (2024)
	Rhein induces apoptosis of AGS and MGC803 cells by regulating the Ras/PI3K/AKT and p38/MAPK signaling pathway	Wan et al. (2024)
	The serine protease CORIN promotes progression of GC by mediating the ERK1/2 MAPK pathway	Hong et al. (2024)
	PRSS23 induces GC stem cell apoptosis and inhibits growth of GC via the MKK3/p38 MAPK-IL24 pathway	Xiong et al. (2024)
GSK3	β-Ionone enhances the inhibitory effects of 5-FU on the proliferation of GC cells by the GSK-3β signaling pathway	Wang et al. (2024a)
	Celastrol impairs tumor growth by modulating the CIP2A-GSK3β-MCL-1 axis in GC cells	Wu et al. (2023a)
	TRPC3 promotes tumorigenesis of GC via the CNB2/GSK3β/NFATc2 signaling pathway	Lin et al. (2021)
CLK	The CLK inhibitor SM08502 induces anti-tumor activity and reduces Wnt pathway gene expression in gastrointestinal cancer models	Tam et al. (2020)

Phosphorylation plays an important role in the occurrence, development, invasion, and drug resistance of GC. Dysregulation of phosphorylation of many oncogenes and tumor suppressor genes is one of the key mechanisms underlying the progression of GC. Studying the abnormal phosphorylation phenomenon in GC can help deepen our understanding of its pathological process and provide new ideas for developing targeted treatment plans.

### 2.3 Acetylation

**FIGURE 4:**
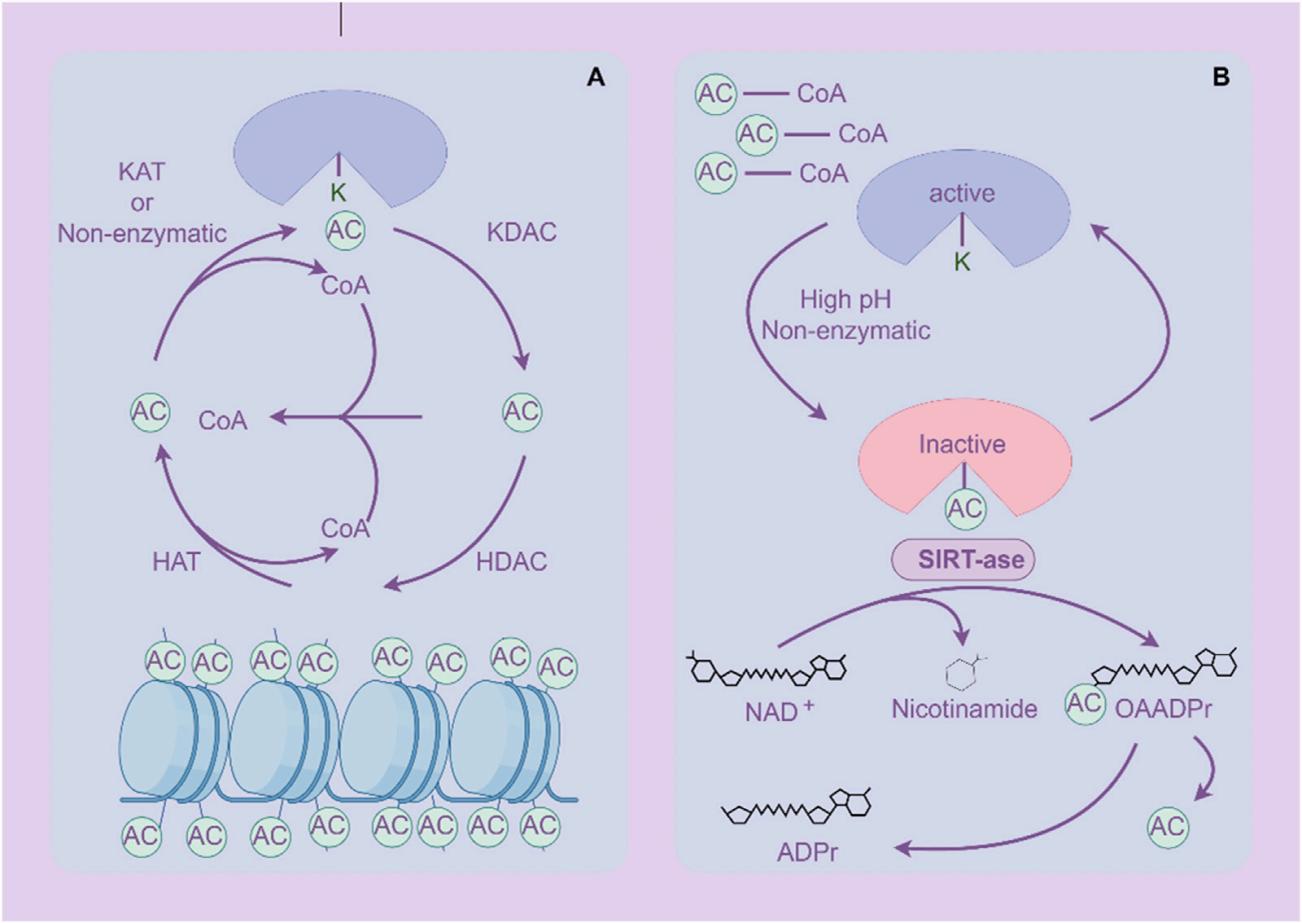
Acetylation and deacetylation processes of proteins. **(A)** Histone protein de/acetylation Process (HDACs family). **(B)** Protein de/acetylation Process (SIRTs family). The figure was drawled by Figdraw (www.figdraw.com/#).

Acetylation is one of the important forms of PTM of proteins, which refers to the addition of acetyl groups (CH3CO) to amino acid residues in proteins, especially lysine residues (Shvedunova and Akhtar, 2022) (Figure 4). Zinc (Zn2+)-dependent histone deacetylases (HDACs) are classified into four major classes: class I (HDACs 1, 2, 3, and 8), class II (HDACs 4, 5, 6, 7, 9, and 10), and class IV, which includes only HDAC11 (Shvedunova and Akhtar, 2022). The class III deacetylases cover the NAD-dependent deacetylases SIRT1–7 (Shvedunova and Akhtar, 2022). Acetylation not only regulates the structure and function of proteins, but also extensively participates in important biological processes such as gene expression, chromatin remodeling, cell cycle regulation, and metabolism (Dang and Wei, 2022; Li and Seto, 2016). Acetylation abnormalities play a crucial role in the occurrence and development of GC (Badie et al., 2022). Histone acetylation is the most common form of acetylation that regulates gene expression. Histones are the core components of chromatin, and by regulating their acetylation levels, the structure of chromatin can be altered, thereby affecting gene expression (Geffen et al., 2023). Acetylation of histones is usually associated with gene activation, which enhances chromatin openness and makes transcription factors more likely to bind to DNA, initiating gene transcription (Zaib et al., 2022). Acetylation not only acts on histones, but also affects the function of various non histone proteins, altering their stability, subcellular localization, interactions, and activity (Narita et al., 2019).

Histone acetyltransferases (HATs) are key regulatory factors in acetylation modification, which can enhance their function by adding acetyl groups to proteins (White et al., 2024). In GC, overexpression of HAT promotes acetylation of histones and non-histones, activating the expression of tumor related genes (Jie et al., 2020; Guo et al., 2022).

Histone deacetylases (HDACs) are important inverse regulators of acetylation modification, inhibiting their function by removing acetyl groups from proteins (Li and Seto, 2016). HDACs are highly expressed in GC, leading to deacetylation of histones and non-histones, and inhibiting the expression and function of tumor suppressor genes (Lin et al., 2023; Jenke et al., 2024). HDAC inhibitors, as a potential anti-cancer treatment, have been applied in the treatment of GC (Jenke et al., 2024). By inhibiting HDACs, the expression of tumor suppressor genes can be restored, inducing apoptosis and differentiation of cancer cells (McClure et al., 2018).

The Sirtuins family is a homolog of yeast chromatin silencing signal regulator 2, which is an NAD+- dependent three class histone deacetylase widely distributed in the body (Nassir, 2022). This family influences the occurrence and development of tumor cells through various pathways, such as regulating gene stability, inflammatory response, cellular stress, apoptosis, energy metabolism of GC cells, and altering the tumor microenvironment (Lagunas-Rangel, 2024; Poniewierska-Baran et al., 2022; Yu L. et al., 2024).

Acetylation is associated with the invasion and metastasis ability of GC (Li et al., 2018). Research has shown that E-cadherin is an important molecule that inhibits cell invasion and metastasis, and its expression and function can be regulated by (Zhao et al., 2019; Tanaka et al., 2002). In GC, HDACs inhibit the expression of E-cadherin through deacetylation, leading to reduced intercellular adhesion and enhancing the invasion and metastasis ability of cancer cells (Decourtye-Espiard et al., 2021). The abnormality of acetylation is closely related to the resistance of GC cells to chemotherapy and targeted therapy. The abnormal expression of HDACs may help GC cells evade chemotherapy induced apoptosis by altering the expression of apoptosis related genes (Regel et al., 2012). In addition, changes in acetylation levels of certain transcription factors may also affect the sensitivity of cells to anticancer drugs (Kokate et al., 2018). The documented roles of these proteins in GC are summarized in Table 4.

**TABLE 4:** Roles of (de-)acetylating enzymes in GC.

Enzyme	Brief biological mechanism	References
KAT2A	KAT2A promotes the succinylation of PKM2 to inhibit its activity and accelerate glycolysis of GC	Zhang and Huang (2024)
P300	TWIST1-EP300 accelerates the resistance of GC cells to apatinib by activating the expression of COL1A2	Yu et al. (2022)
KAT5	CircRHOT1 promoted GC progression and suppressed ferroptosis by recruiting KAT5 to initiate GPX4 transcription	Wang et al. (2023b)
HDAC1	HDAC1-TRIP13/DX21 axis promotes the occurrence and development of GC	Zhang et al. (2024)
HDAC2	The interaction between PAICS and HDAC1/2 promotes the occurrence of GC	Huang et al. (2020)
	Valproic acid targets HDAC1/2 and HDAC1/PTEN/Akt signalling to inhibit cell proliferation via the induction of autophagy in GC	Sun et al. (2020a)
HDAC3	SPI1-ZFP36L1-HDAC3-PD-L1 signaling axis coordinates immune escape in GC	Wei et al. (2024)
	HDAC3-dependent transcriptional repression of FOXA2 regulates FTO/m6A/MYC signaling to contribute to the development of GC	Yang et al. (2021c)
	MBD1/HDAC3-miR-5701-FGFR2 axis promotes the development of GC	Zhao et al. (2022)
	HDAC3 promotes GC occurrence through WNT2bmicro/RNA-376c-3p	Zhang et al. (2021b)
	HDAC3 mediates lncRNA-LOC101928316 activation of PI3K Akt mTOR pathway leading to cisplatin resistance in GC	Ren and Tang (2021)
	HDAC3/lncRNA LET/miR-548k signaling axis mediates GC occurrence	Zhang et al. (2021a)
HDAC4	HDAC4 promotes the growth and metastasis of GC through autophagic degradation of MEKK3	Zang et al. (2022)
	HDAC4 controls the sensitivity of GC to cisplatin through the p53-p73/BIK pathway	Spaety et al. (2019)
HDAC5	The SMAD2/miR-4256/HDAC5/p16INK4a signaling axis contributes to GC progression	Wang et al. (2023e)
HDAC6	HDAC6/FOXP3/HNF4α axis promotes gastric intestinal metaplasia	Zhang et al. (2022)
HDAC7	MiR-489 regulates HDAC7 and PI3K/AKT pathways to inhibit the occurrence of GC	Zhang et al. (2020)
SIRT1	Setd2 inhibits the SIRT1/FOXO pathway to promote GC	Feng et al. (2023)
	LINC00862 competitively bound to miR-29c-3p to unleash SIRT1’s tumor-promoting function	Liu et al. (2024b)
	SIRT1/APE1 promotes the viability of GC	Zhao et al. (2023b)
SIRT2	The LINC00152/miR-138 axis facilitates GC progression by mediating SIRT2	Wang et al. (2021b)
SIRT3	NSAID targets SIRT3 to trigger GC cell death	Debsharma et al. (2024)
	LncRNA FENDRR inhibits GC cell proliferation and invasion through miR-421/SIRT3/Notch-1 axis	Ma et al. (2021)

Due to the important role of acetylation in the occurrence and progression of GC, targeted acetylation therapy strategies are becoming a promising anti-cancer pathway. HDAC inhibitors have shown certain anti GC effects by inhibiting HDAC activity, restoring the expression and function of tumor suppressor genes. In addition, other molecules that target acetylation regulation (histone acetyltransferases, HATs) are also expected to become new therapeutic targets. By regulating acetylation levels, cancer cell proliferation can be effectively inhibited, apoptosis can be promoted, and drug resistance can be reduced (Wu et al., 2020; Marmorstein and Zhou, 2014).

### 2.4 Glycosylation

**FIGURE 5:**
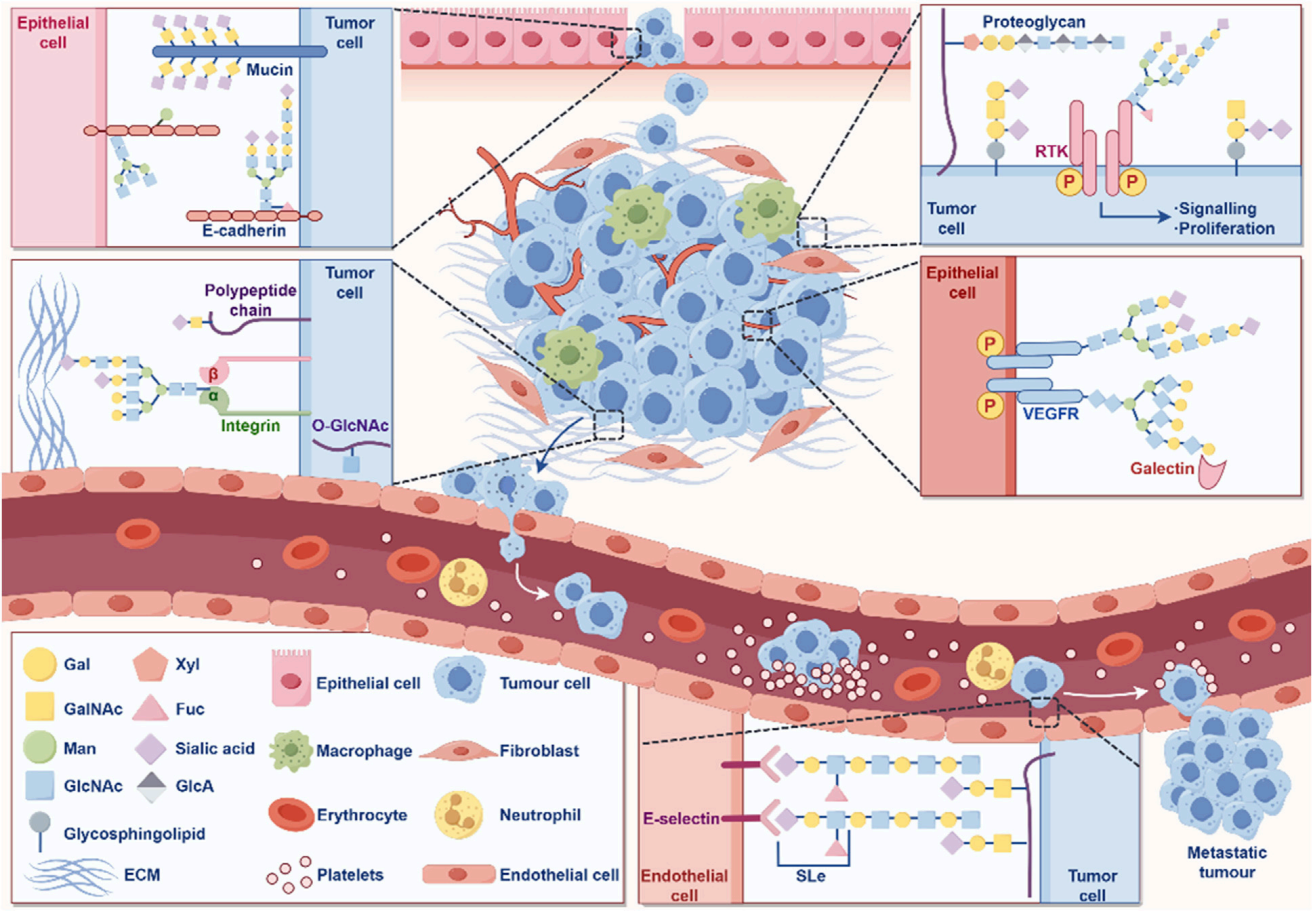
The role of glycosylation in the occurrence and development of cancer. The figure was drawled by Figdraw (www.figdraw.com/#).

Glycosylation is a process in which a protein or lipid is attached to a carbohydrate under the control of an enzyme, aiming to regulate the structure and function of proteins (Eichler, 2019). Glycosylation is one of the important processes in protein PTM. As a common and complex modification, glycosylation plays a crucial role in biological processes such as protein folding, stability, intercellular recognition, and signal transduction (Eichler, 2019). Abnormal glycosylation is closely related to the occurrence and progression of cancer (Figure 5).

In GC, glycosylation abnormalities are manifested in changes in the sugar chain structure and modification patterns of various proteins, which affect the behavioral characteristics of cells and promote the occurrence, progression, and malignant transformation of tumors (Arai et al., 2024; Ferreira et al., 2017). Cancer cells often exhibit abnormally glycosylated sugar chain structures on their surface, including high mannose type and hyper branched structures (Pinho and Reis, 2015; Stowell et al., 2015). These abnormal sugar chains can alter the function of cell membrane receptors, thereby enhancing the activity of signaling pathways, promoting cell proliferation and anti-apoptotic ability (Pinho and Reis, 2015; Stowell et al., 2015). In GC cells, glycosylation modification of EGFR increases its stability on the cell membrane, further activating signaling pathways related to cell proliferation and survival, accelerating tumor growth and malignant progression (Hu et al., 2018). E-cadherin is a key protein that inhibits cell migration, and changes in its glycosylation can affect intercellular adhesion. The abnormal glycosylation of E-cadherin can weaken the adhesion ability between cells and enhance the invasion and metastasis potential of GC cells (Carvalho et al., 2016).

Glycosylation abnormalities are closely related to the expression and activity of multidrug resistance related proteins. The glycosylation of P-gp can enhance its ability to pump chemotherapy drugs, leading to resistance of GC cells to chemotherapy drugs (Liang et al., 2009). Meanwhile, glycosylation modification can alter the expression of surface antigens and affect the recognition of the immune system. GC cells reduce the probability of immune system recognition through abnormal glycosylation, thereby helping them evade immune surveillance, promoting tumor survival and chemotherapy resistance (Sun et al., 2021; Stanczak et al., 2022).The glycosylation process is catalyzed by glycosyltransferases, and the expression and activity of glycosyltransferases in GC often undergo abnormal changes (Pinho and Reis, 2015). GnT-V (N-acetylglucosyltransferase V) is a glycosyltransferase upregulated in GC, which can catalyze the formation of complex sugar chains and is associated with the malignant progression of GC (Huang et al., 2023; Huang et al., 2014). Upregulation of GnT-V can promote the proliferation, invasion, and migration of GC cells, making it a potential therapeutic target (Huang et al., 2014). In summary, glycosylation is crucial for the occurrence and development of GC (Table 5).

**TABLE 5:** Involvement of glycosylation in GC biology.

Sialylation	Fucosylation	Biosynthesis of 1,6 GlcNAc-branched N-glycans	O-linked N-Acetylglucosamine addition
Early Detection of GC (Liu et al., 2024a) GC cell sensitivity to trastuzumab (Duarte et al., 2021)	*In vitro* proliferation, migration, invasion (Yun et al., 2023)	*In vitro* invasion (Zhao et al., 2006)	The development and progression of GC (Jang and Kim, 2016)

Glycosylation plays an important role in the occurrence, progression, metastasis, and drug resistance of GC. We have summarized the specific mechanisms by which various types of glycosylation modifications contribute to the onset and progression of GC (Table 6). Abnormal glycosylation not only alters the proliferation and invasion behavior of GC cells, but is also closely related to the tumor’s resistance to chemotherapy and immunotherapy. By conducting in-depth research on the regulatory mechanisms of glycosylation and developing targeted glycosylation treatment methods, it is expected to provide new ideas and means for the diagnosis, prognosis, and personalized treatment of GC.

**TABLE 6:** Mechanisms of different glycation types in GC research.

Types of glycations	Brief biological mechanism	References
Sialylation	NFB72.3 specifically targets STn sugar chains to reduce the proliferation capacity of GC	Diniz et al. (2023)
	The regulation of glycosyltransferase ST6Gal-I decrease the proliferation of GC cells	Alexander et al. (2020)
	MUC1 and rosmarinic acid can promote apoptosis of GC cells by down-regulating proteoglycosylsialase	Radziejewska et al. (2021)
Fucosylation	FUT11 influences GC occurrence through its involvement in GC pathways such as PI3K-AKT, neuroactive ligand receptors, and MAPK	Huang et al. (2024)
	KIAA1324 promotes the proliferation of GC cells through the interaction between GRP78 and caspase 7	Yun et al. (2023)
	FUT4 promotes GC via MAPK signaling pathway	Aziz et al. (2022)
Biosynthesis of 1, 6 GlcNAc-Branched N-glycans	FUT3 promotes GC cell migration by synthesizing Lea on ITGA6 and GLG1	Wu et al. (2024)
O-Linked N-Acetylglucosamine Addition	O-GlcNAcylation enhances Reticulon 2 protein stability and its promotive effects on GC progression	Wang et al. (2023a)

### 2.5 Methylation

**FIGURE 6:**
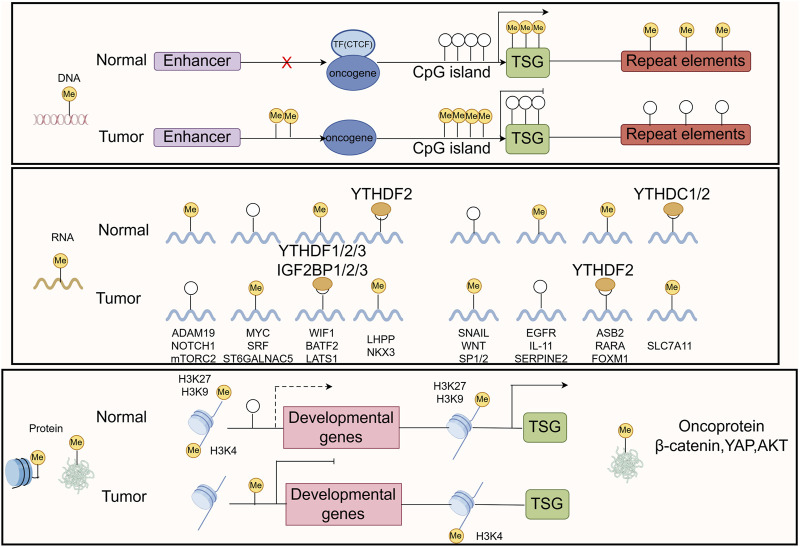
The common mechanisms that cause oncogene/TSG disturbance by methylation remodeling at DNA, RNA, and protein levels are recapitulated in the boxes. The figure was drawled by Figdraw (www.figdraw.com/#).

Methylation is a form of PTM of proteins, particularly DNA and histone methylation, which plays a crucial role in gene expression regulation (Dai et al., 2021; Mattei et al., 2022; Yang B. et al., 2021). Methylation affects the transcriptional activity of genes, the structure of DNA, and the state of chromatin by adding methyl groups (-CH3) at specific base positions (Moore et al., 2013). Methylation remodeling of DNA, RNA, histone, and nonhistone proteins contributes to tumor initiation and progression (Figure 6). In GC, abnormal methylation patterns are closely related to the occurrence, development, invasion, and drug resistance of tumors (Qu et al., 2013; Zeng et al., 2017).

This abnormal methylation leads to the inactivation of tumor suppressor genes, inhibiting functions such as cell cycle regulation, DNA repair, and apoptosis, thereby promoting the proliferation and survival of tumor cells (Wang F. et al., 2022; Zhang N. et al., 2023; Mo et al., 2024). Research has found that common DNA methylation changes in GC tissue are associated with patient prognosis (Usui et al., 2021; Sogutlu et al., 2022), therefore, targeted DNA methylation therapy strategies are considered to have potential clinical application value. Histone methylation plays an important role in regulating gene transcription, chromatin structure, and gene expression. The methylation status of histones H3 and H4 can affect the biological behavior of tumor cells (Audia and Campbell, 2016; Michalak et al., 2019; Liu et al., 2023). Abnormal histone methylation patterns may lead to the inactivation or abnormal expression of tumor related genes, thereby promoting the occurrence and progression of GC (Michalak et al., 2019). The abnormal expression of histone demethylase may be related to the malignant characteristics of GC (Li et al., 2023a; Dong et al., 2023).

In the microenvironment of GC, abnormal methylation can regulate the function of tumor associated macrophages (TAMs) and other immune cells, thereby affecting the tumor’s immune escape ability (Mittelstaedt et al., 2021; Li Y. et al., 2024). Tumor cells evade immune system surveillance and promote cancer progression by altering the phenotype and function of immune cells. The methylation status of drug metabolism related genes in GC cells may affect the tumor’s sensitivity to chemotherapy drugs. Abnormal methylation of some genes can lead to tumor cells developing resistance to chemotherapy drugs, affecting treatment efficacy (Wu Q. et al., 2021; Nagaraju et al., 2021).

Methylation plays an important role in the occurrence, development, invasion, and drug resistance of GC. Abnormal methylation of DNA and histones leads to the inactivation of tumor suppressor genes, promoting the proliferation and survival of cancer cells. Meanwhile, methylation changes are closely related to the tumor microenvironment and drug resistance. By conducting in-depth research on the regulatory mechanisms of methylation and developing targeted methylation therapy methods, it is expected to provide new ideas for early diagnosis, prognosis evaluation, and personalized treatment of GC.

### 2.6 Lactylation

**FIGURE 7:**
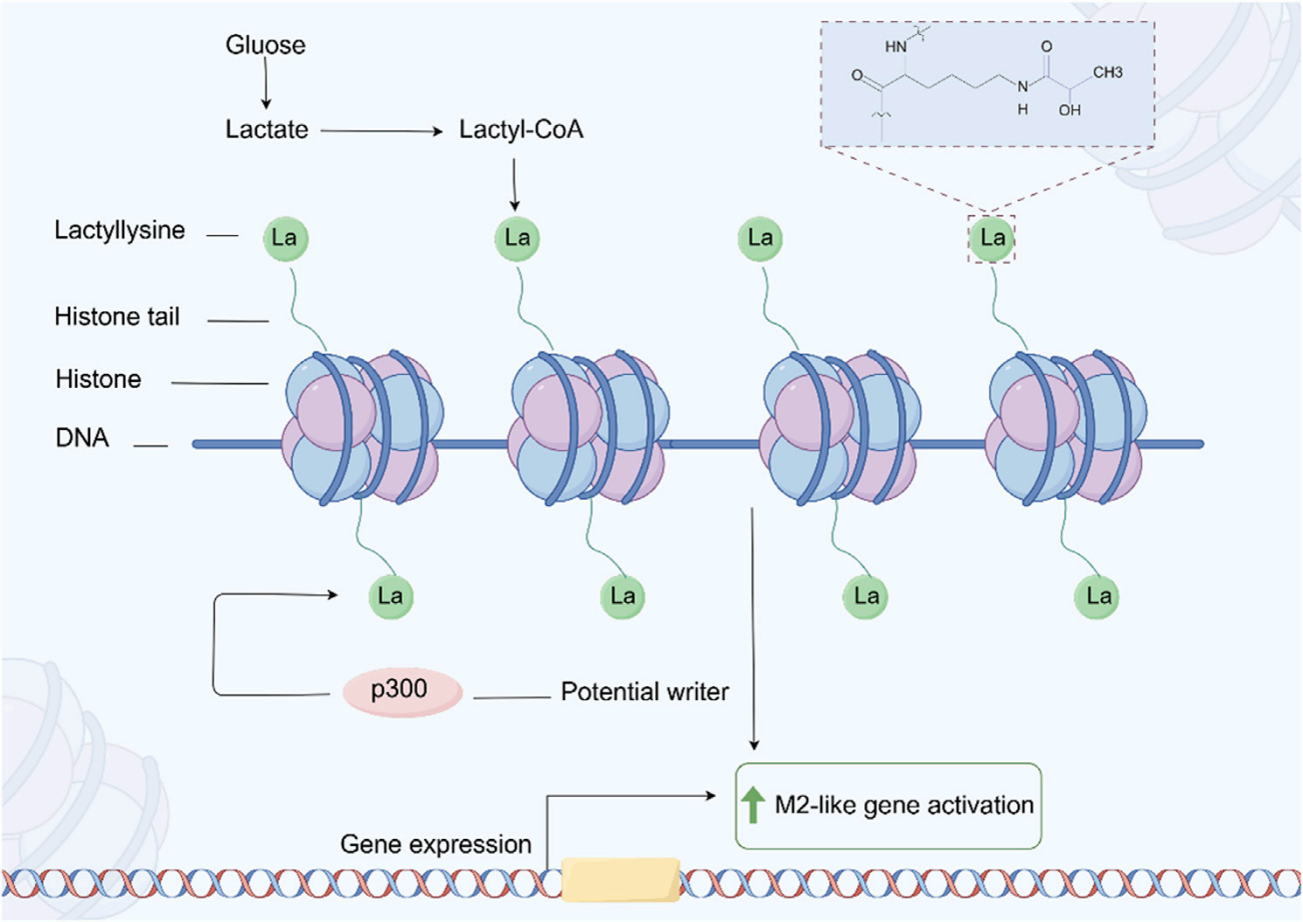
The process of protein Lactylation. The figure was drawled by Figdraw (www.figdraw.com/#).

Lactation is a newly discovered PTM of proteins in recent years, which refers to the covalent addition of lactate molecules (-C3H6O3) to lysine residues in proteins (Fan et al., 2023) (Figure 7). This modification plays an important role in cellular metabolism, signal transduction, and gene expression regulation (Zhang D. et al., 2019). In tumor cells, due to the increased metabolic demand, there is usually a phenomenon of enhanced glycolysis, known as the Warburg effect, which leads to an increase in lactate production (Zhang D. et al., 2019). Tumor cells regulate the functions of various proteins through lactylation, thereby adapting to changes in the tumor microenvironment and promoting cell growth and proliferation (Xie et al., 2023; Qu et al., 2023). Lactation may affect the energy metabolism of tumor cells by regulating the activity or stability of metabolism related enzymes. This modification can increase the flexibility of metabolic pathways and help tumor cells survive under low oxygen and nutrient deficient conditions (Yang H. et al., 2023; Dai et al., 2024; Yang W. et al., 2021).

In GC, the increase in lactate may enhance the migration ability of cancer cells by regulating the reorganization of the cytoskeleton and the expression of intercellular adhesion molecules (Zhao et al., 2024; Li Z. et al., 2024). Studies have shown that lactylation may affect signaling pathways related to cell adhesion and migration (Wang J. et al., 2022). The drug resistance of GC cells in chemotherapy and targeted therapy is often related to metabolic reprogramming and changes in intracellular signaling pathways (Bin et al., 2021). Lactic acid may promote cancer cell tolerance to treatment by regulating signaling pathways related to drug resistance. Lactic acid modification of certain key proteins may affect drug targeting, leading to increased excretion of chemotherapy drugs in cancer cells or loss of target function (Yu X. et al., 2024; Zha et al., 2024; Chen H. et al., 2024).

Lactic acid plays an important role in the metabolic regulation, gene expression, tumor microenvironment, and drug resistance of GC. Lactic acid promotes the development and malignant progression of GC by regulating protein functions related to metabolism, cell proliferation, and immune escape. In depth research on the mechanism of lactylation and the development of treatment strategies targeting lactylation are expected to provide new ideas for the early diagnosis, treatment, and prognosis evaluation of GC.

### 2.7 SUMOylation

SUMOylation (Small Ubiquitin like Modifier) refers to a PTM that covalently attaches SUMO proteins to lysine residues of target proteins (Han et al., 2018). Similar to ubiquitination, SUMOylation regulates various cellular processes by altering protein stability, activity, subcellular localization, or interactions with other proteins (Wei et al., 2023; Hu et al., 2021; Wu et al., 2023b). In recent years, the role of SUMOylation in tumor biology has gradually received attention, especially in GC, where abnormal SUMOylation is closely related to the occurrence, development, invasion, and drug resistance of tumors (Xie et al., 2020; Seeler and Dejean, 2017).

The increase or decrease of SUMOylation can promote the occurrence and development of GC by inhibiting or enhancing the expression of specific genes (Zhao Y. Q. et al., 2023; Wang T. et al., 2023). SUMOylation can also regulate gene expression by binding to transcription factors (Tian et al., 2024). In GC cells, abnormal SUMOylation may lead to uncontrolled cell cycle and promote abnormal proliferation of cancer cells (Fang et al., 2017; Gu et al., 2024).

SUMOylation also plays an important role in the process of DNA damage repair. By regulating the SUMOylation status of proteins involved in DNA repair, it can affect the efficiency of DNA repair and genomic stability (Zhang F. L. et al., 2023). In GC, DNA repair defects are closely related to tumor development, and abnormal SUMOylation may lead to the accumulation of DNA damage, promoting the occurrence of cancer (Zhang M. et al., 2019). SUMOylation can affect the migration and invasion ability of GC cells by regulating the functions of cytoskeleton related proteins and cell adhesion molecules (Wang Q. et al., 2021; Liu et al., 2021). The SUMOylation of intercellular adhesion molecules and integrins may alter their functions, promoting cell detachment from the primary tumor and migration to distant organs. SUMOylation may also affect the progression of GC by regulating the interaction between tumor cells and the surrounding microenvironment (Gu et al., 2023). The low oxygen state in the tumor microenvironment can regulate the stability of hypoxia inducible factors (HIFs) through SUMOylation, promoting GC angiogenesis and tumor cell survival under low oxygen conditions (Filippopoulou et al., 2020; Zhou et al., 2021). In addition, SUMOylation plays an important role in the drug resistance of GC. SUMOylation may affect the efficacy of chemotherapy drugs by regulating proteins involved in drug metabolism, leading to drug resistance in GC cells (Gu et al., 2024; Huang et al., 2022a).

SUMOylation, as a key protein PTM, plays multiple roles in the occurrence, development, invasion, and drug resistance of GC. By regulating the SUMOylation status of transcription factors, cell cycle proteins, DNA repair related proteins, and cell migration related factors, GC cells can acquire the ability to proliferate, invade, and resist treatment. Therefore, in-depth research on the specific mechanism of SUMOylation in GC and the development of targeted SUMOylation treatment methods will provide new ideas for the treatment of GC.

### 2.8 PTM crosstalk

PTM crosstalk refers to the phenomenon of mutual influence between different types of PTM, which plays an important role in regulating protein function, stability, and interaction networks (Huang et al., 2019; Geffen et al., 2023; Cutler et al., 2021). PTM crosstalk can occur in both intraprotein and interprotein contexts, involving the same or different types of modifications. Regardless of the specific mechanisms, PTM crosstalk can orchestrate complex interactions among various PTM, influencing protein functions, signaling pathways, and the regulation of protein networks in tumorigenesis. This interplay plays a crucial role in the development and progression of tumors, highlighting the profound impact of PTM on cellular fate and pathological processes (Wang W. et al., 2024; Li et al., 2023b; Wu et al., 2019; Hernandez-Valladares et al., 2019).

In GC, common PTM include phosphorylation, acetylation, methylation, and ubiquitination, and the interactions between these modifications may significantly affect protein activity. PTM crosstalk also plays an important role in cellular signaling pathways. Taking the NF - κB signaling pathway as an example, this pathway plays a crucial role in the development of various tumors. The activity of NF - κB is regulated by various PTM such as phosphorylation, acetylation, and ubiquitination. Research has shown that acetylation modification of NF - κB can enhance its transcriptional activity, while phosphorylation may affect its transcriptional activity in the nucleus by altering its affinity for binding proteins. In addition, ubiquitination modification of NF - κB can promote its degradation, thereby regulating its stability in cells. These complex PTM interactions enable NF - κB to flexibly regulate its function in different cellular environments (Ito, 2007). In the RAS/MAPK pathway, KRAS and other signaling mediators are influenced by various PTM, including phosphorylation, ubiquitination, farnesylation, proteolysis, methylation, and palmitoylation (Ahearn et al., 2011; Laude and Prior, 2008). Many signaling mediators in the TGF - β pathway are widely influenced by PTM, including phosphorylation and ubiquitination, which are crucial for initiating and regulating signal transduction to the nucleus (Xu et al., 2016). The activation/inactivation of tumor suppressor gene p53 function is regulated by various PTM, including phosphorylation, ubiquitination, acetylation, and methylation (Bode and Dong, 2004; Dai and Gu, 2010).

As an emerging field of PTM research, the study of PTM crosstalk in cancer is still somewhat blank. Therefore, understanding the mechanism of PTM crosstalk is particularly important for developing new therapeutic strategies, especially when targeting specific signaling pathways or regulating protein functions, which can provide new ideas and methods for precision medicine.

## 3 Discussion

### 3.1 Limitations of PTM in GC research

Although PTM play a crucial role in cell biology, there are still significant limitations to current research on their use in GC. PTM such as ubiquitination, phosphorylation, acetylation, glycosylation, methylation, lactylation, and SUMOylation regulate protein stability, activity, and interactions, but how these modifications alter tumor behavior in GC has not been fully elucidated. Most of the research has focused on genomic and epigenetic regulation, while there is relatively little research on the detailed role and crosstalk of PTM in GC. he complexity of PTM mechanisms makes target selection and drug design challenging, especially in cases where significant differences exist between cancer subtypes and individuals, limiting the broad applicability of PTM-targeted therapies. Additionally, the high cost and complexity of research technologies restrict the widespread clinical application of these methods. The challenge of individualized treatment is another critical issue, as variations in PTM across different patients may lead to differential drug responses, making precise treatment difficult. PTM-targeted therapies may influence off-target genes, potentially inducing side effects or affecting normal cell functions. Furthermore, the prolonged use of PTM-targeted drugs may lead to drug resistance, impacting the long-term effectiveness of treatment. These limitations necessitate further scientific research and technological advancements to overcome these challenges and enhance the clinical utility of PTM-targeted therapies. Filling this gap is expected to reveal new biological mechanisms and potential therapeutic targets.

### 3.2 The function and role of PTM and crosstalk in GC

In GC, PTM (ubiquitination, phosphorylation, acetylation, glycosylation, methylation, lactylation and SUMOylation, etc.) affect biological processes by regulating protein stability, activity, and interactions. For example, ubiquitination regulates protein degradation (Sun T. et al., 2020), phosphorylation participates in the activation of key signaling pathways (Agashe et al., 2022; Luo et al., 2020; Ebert et al., 2022), acetylation and methylation affect gene expression, while glycosylation plays a role in intercellular signaling (Xu and Wan, 2023; Bao and Wong, 2021; Ramaiah et al., 2021; Jarrold and Davies, 2019; Li et al., 2020; Locke et al., 2019). Lactylation is associated with metabolic reprogramming (Sun L. et al., 2022; Lv et al., 2023), while SUMO modification is associated with tumor drug resistance and progression (Chang and Yeh, 2020). In addition, the crosstalk between different modifications makes the regulatory mechanism more complex, which affects protein function and tumor cell behavior, especially playing an important role in the invasion and metastasis of GC.

The complexity of PTM is reflected in the interplay and crosstalk between different types of PTM. Various modifications such as ubiquitination, phosphorylation, and acetylation play a critical role in regulating tumor cell processes, including growth, migration, invasion, and immune evasion. For instance, the interplay between phosphorylation and ubiquitination can enhance kinase activity, promoting tumor cell survival and dissemination (Cutler et al., 2021; Barbour et al., 2023). Additionally, acetylation and SUMOylation contribute to the regulation of protein stability and function. PTM crosstalk not only affects the individual roles of specific PTM but also integrates multiple signaling pathways to control the complex behaviors of tumor cells (Barbour et al., 2023). These mechanisms play a pivotal role in the progression and drug resistance observed in GC, where tumor cells exploit the PTM network to evade therapeutic inhibition. Therefore, a deeper understanding of PTM crosstalk mechanisms is essential for the development of more precise and effective targeted therapies for GC.

As research progresses, PTM-targeted therapies are increasingly being recognized as a crucial strategy in the treatment of GC, aiming to disrupt abnormal signaling pathways in tumor cells through targeted modifications. For instance, drugs targeting phosphorylation kinases or ubiquitination-regulated proteins can interfere with these modifications to inhibit tumor cell proliferation and migration (Wang et al., 2020; Su et al., 2022).

### 3.3 Implications for future cancer research

In GC research, PTM and their crosstalk mechanisms play critical roles in regulating various biological processes in tumor cells. Despite significant advances, there remain substantial challenges and limitations. Current studies primarily focus on certain PTM types, such as phosphorylation and ubiquitination, while the functional mechanisms of less-studied PTM, such as glycosylation and lactylation, are still underexplored. With ongoing research, more novel PTM are being identified, yet studies on these modifications remain at the preliminary stages of screening and validation, with limited clinical applicability. Moreover, the dynamic nature of PTM and their intricate networks within the tumor microenvironment add layers of complexity to the selection of therapeutic targets and the development of effective treatment strategies. Many PTM-targeted drugs face challenges related to target generalization, lacking precise interventions for specific PTM or PTM networks.

Future research should delve deeper into several key areas. First, leveraging high-throughput omics technologies, such as mass spectrometry and single-cell RNA sequencing, to comprehensively characterize the dynamic changes in PTM networks and identify critical modification sites with functional significance in various cellular states (Gillette et al., 2024; Li and Zhan, 2020; Kirsch et al., 2020; Qin et al., 2020). Second, integrating bioinformatics and machine learning approaches to predict and screen effective drugs targeting PTM while optimizing the selectivity and efficacy of existing PTM-targeted therapies. Additionally, research should address the variability of PTM responses among individuals, tumor subtypes, and their microenvironments to design more personalized and adaptable therapeutic strategies (Wang et al., 2023d; Hegde et al., 2020).

Another significant challenge lies in addressing the long-term safety and resistance associated with PTM-targeted drugs. Prolonged use of such therapies may prompt tumor cells to remodel PTM networks, enabling them to evade drug inhibition and develop resistance (Wang Y. et al., 2023; Onglao et al., 2022). Therefore, future efforts should prioritize exploring combination targeting strategies, integrating multiple PTM and diverse biological pathways to enhance therapeutic efficacy and mitigate resistance risks. By adopting these comprehensive strategies, PTM-targeted therapies could more precisely and effectively disrupt the complex biological mechanisms of GC, ultimately improving clinical outcomes for patients.

## 4 Conclusion

Multiple protein PTM mechanisms are closely involved in the occurrence, progression, and treatment tolerance of GC. Ubiquitination affects the proliferation and apoptosis of cancer cells by regulating the degradation of key proteins; Acetylation modification regulates gene expression, especially at the epigenetic level, by affecting the activity of oncogenes and tumor suppressor genes through histone acetylation and deacetylation; Abnormal glycosylation alters the invasiveness and immune escape ability of cancer cells; Methylation is involved in gene silencing and oncogene activation, and is a common epigenetic change in GC; Lactic acid modification, as an emerging research field, may be related to metabolic reprogramming in the tumor microenvironment; Phosphorylation is the core of signal pathway regulation, affecting cell proliferation and survival; SUMOylation plays an important role in cancer drug resistance by regulating protein stability and DNA repair. These modifications together form a complex network for the development of GC and provide multiple potential targets for diagnosis and therapeutic interventions. Overall, these PTM participate in the multifaceted regulation of GC through synergistic or independent pathways, and provide rich potential targets for the development of diagnostic biomarkers and targeted therapy strategies.
